# Collecting and sharing self-generated health and lifestyle data: Understanding barriers for people living with long-term health conditions – a survey study

**DOI:** 10.1177/20552076221084458

**Published:** 2022-03-07

**Authors:** Richard Brown, Lynne Coventry, Elizabeth Sillence, John Blythe, Simone Stumpf, Jon Bird, Abigail C. Durrant

**Affiliations:** 1 Psychology Department, 411646 Northumbria University, Newcastle, UK; 2 Immersive Labs, London, UK; 3 Department of Computer Science, 4919 City University of London, UK; 4 Department of Computer Science, 1980 University of Bristol, UK; 5 Open Lab, 5994 Newcastle University, UK

**Keywords:** Disease, public health, eHealth, personalised medicine, digital health, self-generated health and lifestyle data, psychology, risk perceptions, health communications

## Abstract

**Background:**

The growing popularity of collecting self-generated health and lifestyle data presents a valuable opportunity to develop our understanding of long-term health conditions and improve care. Barriers remain to the effective sharing of health and lifestyle data by those living with long-term health conditions which include beliefs around concepts of Trust, Identity, Privacy and Security, experiences of stigma, perceptions of risk and information sensitivity.

**Method:**

We surveyed 250 UK adults who reported living with a range of long-term health conditions. We recorded data to assess self-reported behaviours, experiences, attitudes and motivations relevant to sharing self-generated health and lifestyle data. We also asked participants about their beliefs about Trust, Identity, Privacy and Security, stigma, and perceptions of risk and information sensitivity regarding their health and lifestyle data.

**Results:**

Three-quarters of our sample reported recording information about their health and lifestyle on a daily basis. However, two-thirds reported never or rarely sharing this information with others. Trust, Identity, Privacy and Security concerns were considered to be ‘very important’ by those with long-term health conditions when deciding whether or not to share self-generated health and lifestyle data with others, with security concerns considered most important. Of those living with a long-term health condition, 58% reported experiencing stigma associated with their condition. The greatest perceived risk from sharing with others was the potential for future harm to their social relationships.

**Conclusions:**

Our findings suggest that, in order for health professionals and researchers to benefit from the increased prevalence of self-generated health and lifestyle data, more can be done to address security concerns and to understand perceived risks associated with data sharing. Digital platforms aimed at facilitating the sharing of self-generated health and lifestyle data may look to highlight security features, enable users to control the sharing of certain information types, and emphasise the practical benefits to users of sharing health and lifestyle data with others.

## Introduction

There are approximately 19 million people currently living with a long-term health condition (LTHC) in the UK.^1^ The Department of Health in England has defined a LTHC as ‘one that cannot currently be cured but can be controlled with the use of medication and/or other therapies’.^2^ With the prevalence of LTHCs expected to rise in the coming decades,^3^ it is essential that we develop strategies to enable both healthcare systems and individual patients to better manage health and care in the UK.

One solution to help manage the increasing prevalence and cost of long-term care is the use of eHealth, defined as the enhanced use of digital information and communication technology (ICT) in healthcare.^4^ The increasingly ubiquitous nature of technology has meant that eHealth and related tools can provide a convenient means for collecting and sharing objective patient-generated data in real time.^5^ For example, the use of wearable devices to track and monitor health and wellbeing has risen significantly in recent years.^6^ Widening the channels through which health data is collected and shared between patients and healthcare professionals (HCPs) may have particular significance for those living with LTHCs, enabling the best use of the infrequent and limited contact time that such patients typically have with relevant HCPs.^7^ For example, patients with conditions such as diabetes report having as little as three hours of contact time a year with HCPs, with the majority of their health needs being self-managed.^7^ Benefits to patients from sharing *self*-generated health and lifestyle data with others include the potential for greater autonomy and better overall health outcomes. ‘Self-generated health and lifestyle data’ covers a broad range of data types from a varied list of data sources. This may include handwritten records of information about sleep, diet or use of medication, as well as encompassing information collected via wearable medical devices such as heart rate, blood sugar and levels of physical activity. Sharing such health and lifestyle data with others has been linked to better health management, due to those who share being more likely to implement better self-care than those who do not.^8^ In a study of patients with epilepsy, the perceived benefits of sharing health data with others included gaining a better understanding of seizures and learning more about symptoms and treatment.^8^ Those who share via community platforms such as PatientsLikeMe perceive the greatest benefits to sharing as having the opportunity to learn about their symptoms and to understand the side effects of their treatment.^9^ Furthermore, a recent study of patients with rheumatoid arthritis found that collecting and sharing self-generated health data led to consultations being more focused around their actual data, making patients feel that they are receiving more personalised care.^10^ In the same study, the perceived benefits to public health surveillance and research from the collection and sharing of self-generated health data were reported to be the identification of disease patterns and long-term trends that would otherwise be concealed amongst the daily fluctuation of symptoms.^10^ Increasing the scope and availability of self-generated health and lifestyle data may allow the application of big data practices to public health in order to conduct exploratory analyses to identify patterns across previously separated disciplines (such as among public health research, healthcare, biology, ecology and demography).^11^ Big data practices can be defined as the structured, sophisticated and rapid analysis of large complex data sets.^11,12^ This may help to provide a multidisciplinary approach to understanding health phenomena beyond the capabilities of single disciplines. The benefits of this big data approach may be to optimise the delivery of care for individual patients by providing information to support decision making and care planning by HCPs. This will require greater levels of sharing across multiple data points to facilitate appropriate and necessary research.^13^ For both the data provider and others (through results of public health research) to fully benefit from such developments, the safe and effective sharing of health and lifestyle data with others should be encouraged.^14^

It is noted that while the use of self-generated health and lifestyle data for improved care is presented as a patient-centred, low-cost health solution, it has the potential to add to the increasing workload of HCPs. If the information is not available in an accessible and appropriate manner, it can require excessive time to analyse or make sense of the data provided. It is especially important to be mindful of this fact at a time when HCPs are under immense pressure in response to the COVID-19 pandemic. Potential concerns have been raised about the reliability and accuracy of patient-generated data presented to HCPs.^15,16^ Research suggests that HCPs themselves have doubts about the reliability of the health technology available to the general population.^17^ These concerns may create difficulties for HCPs when they are required to judge the utility of data provided during health consultations. This highlights the need for clarity surrounding how best to integrate self-generated health and lifestyle data into the delivery of care.

Additionally, it is important to appreciate the technological preferences and abilities of individual patients before asking them to actively collect, monitor, share and manage their health data. This may help to avoid burdening individual patients with unwelcome responsibility.^18^ Though collecting health data can sometimes be conducted passively by digital devices, research into the perspectives of those living with multiple LTHCs found that, in some cases, managing self-generated health and lifestyle data can become a time-consuming burden that exacerbates the struggles of existing illnesses.^19^ Therefore, supporting patients to effectively share self-generated health and lifestyle data requires close consideration of patient technological preferences and must be delivered without over burdening the data gatherers or HCPs.

It is suggested that in order to benefit from large quantities of self-generated health and lifestyle data, people with LTHCs should be supported and encouraged to collect and share information about their health.^14^ Despite the potential benefits of sharing self-generated health and lifestyle data with others, a number of barriers have been identified that prevent the acceptance of these data-sharing practices. For instance, as electronic health data become increasingly integrated into healthcare systems, there is an increased potential for privacy breaches and misrepresentation, negatively influencing end-user trust^20^ In this research we will discuss concerns surrounding concepts of Trust, Identity, Privacy and Security (TIPS) adopted from research into privacy and security perspectives.^21^ TIPS concerns play an important role in facilitating sharing of health data via technology applications.^21–24^ For example, trust has been identified as a key factor for increasing the likelihood of patients sharing health information for the purpose of participating in research, whereas lack of trust has been shown to decrease willingness to share.^25^ By identity issues we refer to individual concerns about identifiers that are attributed to a person that may be used to interact with both physical and digital worlds. For example, identifiers in the physical world may refer to one's name, location, self-representations and factors relevant to someone's face-to-face interactions. Digital identify refers to identifiers relevant to one's personal data and online presence. Privacy and security concerns refer to issues surrounding the ability to maintain the private and secure storage of personal data and information. Such concerns have been found to be negatively associated with patient willingness to share health information with others.^20^ In recent years, there has been increasing interest in the role that such TIPS concerns play in the sharing of health and lifestyle data among those with LTHCs.^26^ For example, in a qualitative study of the experiences of patients with HIV (an example of an LTHC that is associated with experiences of stigma),^21,27^ TIPS concerns were found to be central to perceptions of sharing health data with others.^17^

In addition to TIPS concerns, information sensitivity has been found to impact both privacy concerns and willingness to share data with others.^20,27^ Perceived sensitivity of information has been suggested to be a key barrier to the sharing of health and lifestyle data with others, yet it is difficult to define and measure.^28^ Furthermore, fears and perceived risks surrounding the unwanted disclosure of health data may cause some individuals to expect harmful consequences as a result of sharing information they deem to be sensitive.^20^ When deciding whether or not to share health information with others, individuals may first weigh up the benefits to sharing against the perceived risks.^28^

Finally, people who live with conditions that are typically associated with stigma may anticipate potential discrimination, harm or negative labels when considering whether or not to share health information with others.^29^ Stigma can be both internal (felt stigma or self-stigmatisation) or enacted (external or discrimination) experiencing unfair treatment from others.^30^ Both internal and enacted stigma can influence the way in which patients develop trust and may choose to share their self-generated health and lifestyle data.^26,31–35^ A number of health conditions are frequently associated with experiences of stigma,^36^ such as living with HIV,^21,37^ mental health problems,^36,38^ and chronic pain.^39^ People living with LTHCs who anticipate stigma associated with their condition(s) may be more reluctant to share their health data which could potentially prevent them from receiving an appropriate level of care.^31,40^ Our specific interest in stigma aligns with the objectives of the broader research programme. This survey study is conducted as part of a UK EPSRC funded programme (‘INTUIT: Interaction Design for Trusted Sharing of Personal Health Data to Live Well with HIV’, 2020)^41^ examining TIPS concerns around the sharing of self-generated health and lifestyle data primarily among those living with HIV, but also looks to investigate TIPS concerns among those living with a range of other LTHCs. The INTUIT project aims to identify TIPS concerns and to design tools that remove the barriers to collecting and sharing self-generated health and lifestyle data in order to improve the health and well-being of stigmatised populations.

Given the increasing prevalence of LTHCs, and the potential benefits of utilising self-generated health and lifestyle data, it is paramount that health systems understand the attitudes and perceptions of those living with LTHCs in order to promote the beneficial sharing of health and lifestyle data. To address this, we have conducted a quantitative survey of the attitudes and behaviours of people living with a range of LTHCs with respect to the sharing of self-generated health and lifestyle data with others. This study forms part of a wider programme of research exploring TIPS concerns around self-generated data sharing to inform supportive and trusted technology designs for managing LTHCs (INTUIT).^41^ Our study aim was threefold: (i) to identify the extent to which TIPS concerns relevant to the sharing of self-generated health and lifestyle data with others are reported by those living with a range of LTHCs; (ii) to explore if perceptions of risk and information sensitivity are associated with data sharing perceptions and behaviours, attitudes towards sharing, and TIPS concerns; and (iii) to examine the impact of stigma by identifying behavioural and perceptual differences between those who report experiences of stigma and those who do not, and by exploring the relationship between perceived stigma and the sharing of self-generated health and lifestyle data. Finally, we address the role that these insights may play in designing future digital platforms for enabling trusted, private and secure health data sharing in a range of settings.

## Method

Our study was approved by the Department of Psychology Ethics Committee at Northumbria University (ethical approval number 26581). Our measures, predictions and study protocol are registered with the Open Science Framework (osf.io/h3mjv/)*.* We surveyed 251 UK participants, aged 18 or above, who reported living with a LTHC. We recruited participants via the surveying platform Prolific as it is a company that offers a high-quality participant pool of research-participant volunteers. We used Prolific's pre-screening criteria, which allowed us to ensure that only those who had self-reported living with a LTHC had access to our survey. An a priori power analysis indicated that a sample size of 211 was required to detect a small-to-medium effect of .2 for a bivariate correlational analysis with a power of .90 and an alpha of .05. This would allow us to conduct an independent correlational analysis across measures of perceived stigma, TIPS concerns, willingness to share with others, perceived stigma and sharing behaviours. Therefore, to account for the possibility of missing data, our final recruited sample size was 251 participants. We conducted data quality checks by ensuring that responses for age and gender in our survey were consistent with responses given on participants’ Prolific profiles. Attention checks ensured that participants took a minimum of 8 min to complete the survey. We excluded one participant because they took over 3 h (192 min) to complete the survey. Therefore, our final sample contains 250 participants: 166 females, 80 males, two non-binary and two who preferred not to report their gender, aged 18–77 years (mean age = 39.20 years, SD = 14.78). See Tables 1 and 2 for full participant details.

**Table 1.** Sample characteristics for age, gender, ethnicity and sexual orientation.

	Category	Number (*N* *=* *250*)	Percentage of sample
Age	18–34	108	43.2
* *	35–49	77	30.8
* *	50–64	50	20.0
* *	65 +	15	6.0
Gender	Male	80	32.0
	Female	166	66.4
	Non-binary	2	.8
	Prefer to self-describe	2	.8
	Prefer not to say	0	0
Ethnicity	White – English/Welsh/Scottish/Northern Irish /British	220	88.0
	White – Irish	3	1.2
	White – Gypsy or Irish Traveller	1	.4
	White – Any other White background	10	4.0
	Mixed/Multiple ethnic groups – White and Black Caribbean	2	.8
	Mixed/Multiple ethnic groups – White and Asian	2	.8
	Asian/Asian British – Indian	2	.8
	Asian/Asian British – Bangladeshi	1	.4
	Asian/Asian British – Chinese	3	1.2
	Asian/Asian British – Any other Asian background	3	1.2
	Black/ African/Caribbean/Black British – African	1	.4
	Arab	1	.4
	Any other ethnic group	1	.4
	Prefer not to say	0	0
Sexual orientation	Straight or Heterosexual	204	81.6
	Gay or Lesbian	12	4.8
	Bisexual	28	11.2
	Other sexual orientation	4	1.6
	Prefer not to say	2	.8

**Table 2.** Reported frequencies for LTHCs and primary LTHCs.

LTHC	Number of reports	Percentage of total LTHCs reported	Percentage of sample	Number reported as primary LTHC	Percentage of sample reported as primary LTHC
Acne	16	2.4	6.4	1	.4
Alcohol problems	5	0.7	2.0	0	0
Anorexia or bulimia	2	0.3	0.8	0	0
Anxiety	87	12.9	34.8	13	5.2
Asthma	46	6.8	18.4	17	6.8
Atrial fibrillation	2	0.3	0.8	2	.8
Bronchiectasis	1	0.1	0.4	1	.4
Cancer	6	0.9	2.4	4	1.6
Cardiovascular disease	3	0.4	1.2	1	.4
Chronic fatigue syndrome	19	2.8	7.6	11	4.4
Chronic kidney disease	3	0.4	1.2	1	.4
COPD	2	0.3	0.8	2	.8
Chronic sinusitis	2	0.3	0.8	1	.4
Chronic tissue disorder	2	0.3	0.8	0	0
Coronary heart disease	2	0.3	0.8	2	.8
Depression	88	13.1	35.2	21	8.4
Diabetes (type 1)	7	1.0	2.8	6	2.4
Diabetes (type 2)	12	1.8	4.8	4	1.6
Diabetes (type not specified)	6	0.9	2.4	5	2.0
Diverticular disease	4	0.6	1.6	0	0
Dyspepsia	4	0.6	1.6	1	.4
Endometriosis	11	1.6	4.4	5	2.0
Epilepsy	5	0.7	2.0	3	1.2
Erectile dysfunction	2	0.3	0.8	0	0
Glaucoma	1	0.1	0.4	0	0
Heart failure	3	0.4	1.2	1	.4
Hypertension	19	2.8	7.6	6	2.4
Incontinence	2	0.3	0.8	1	.4
Inflammatory bowel disease	7	1.0	2.8	5	2.0
Irritable bowel syndrome	30	4.5	12.0	4	1.6
Meniere's disease	3	0.4	1.2	0	0
Mental health condition	35	5.2	14.0	6	2.4
Migraine	31	4.6	12.4	5	2.0
Multiple sclerosis	6	0.9	2.4	5	2.0
Obesity	24	3.6	9.6	2	.8
Osteoporosis	6	0.9	2.4	2	.8
Painful conditions	38	5.7	15.2	14	5.6
Parkinson's disease	1	0.1	0.4	1	.4
Pernicious anaemia	2	0.3	0.8	1	.4
Polycystic ovary	12	1.8	4.8	3	1.2
Prostate disorders	1	0.1	0.4	0	0
Psoriasis/eczema	30	4.5	12.0	4	1.6
Schizophrenia or bipolar disorder	5	0.7	2.0	2	.8
Sexual health condition	2	0.3	0.8	0	0
Stroke/transient ischaemic attack	1	0.1	0.4	1	.4
Thyroid disorder	16	2.4	6.4	8	3.2
Treated constipation	2	0.3	0.8	0	0
Other condition	58	8.6	23.2	31	12.4
Multiple long-term conditions				47	18.8
Total	672	100	268.8	250	100

### Personal information

Participants’ age, gender, ethnicity and sexual orientation were recorded. Participants were asked to confirm that they had a LTHC and to indicate the nature and duration of their condition(s). A dropdown list of LTHCs was presented to participants, taken from recent research into LTHCs in the UK Biobank cohort.^42^ Participants could provide multiple responses or self-describe their LTHC(s). If participants stated that they are living with more than one LTHC, they were asked to indicate which condition they consider to be their ‘Primary LTHC’. If a participant felt that more than one condition is fundamental to their primary health needs, their primary health condition was categorised as ‘Multiple LTHCs’.

### Self-generated health and lifestyle data behaviours

Participants were asked how often they record their own health and lifestyle data and the type of self-generated data they record. Participants indicated the frequency of data collection (selecting ‘never’, ‘when the need arises’, ‘less than once a month’, ‘monthly’, ‘fortnightly’, ‘weekly’ or ‘daily’) for a list of 17 separate categories of self-generated health and lifestyle data developed from previous literature (e.g. ‘effects of medication’, ‘blood pressure’, ‘heart rate’, ‘sleep patterns’, ‘diet’ and including the option to self-describe additional categories).^43^ Participants were asked what method(s) they use to record or monitor their health and lifestyle data, selecting answers from nine predefined methods and devices developed from previous research (e.g. ‘mobile phones’, ‘wearable activity trackers’ and ‘handwritten records’ and including the option to self-describe additional methods; see preregistration document for full questionnaire details (osf.io/h3mjv/).^44^ Participants also indicated how often they share different types of self-generated health and lifestyle data with others, and with whom.

### Experiences of sharing health data

Participants rated how positive and beneficial they have found experiences of sharing self-generated health and lifestyle data with others (rated on a five-point Likert scale from ‘extremely negative/detrimental’ to ‘extremely positive/beneficial’). Participants also rated the extent to which they consider recording their health and lifestyle data helps them to understand their condition(s) and whether they think others can benefit from their data (rated on a five-point Likert scale from ‘not at all’ to ‘a great deal’).

### Motivation for sharing

Participants indicated the extent to which five separate aims motivate them to share health and lifestyle data with others. Participants were asked to what extent they share health and lifestyle data with others in order to improve their own health, to improve the health of others, to receive emotional support from others, to provide emotional support to others, or to receive practical support to help manage their condition. Participants responded by stating the extent of their agreement with five statements about their motivation to share health and lifestyle data with others (rated on a five-point Likert scale from ‘strongly disagree’ to ‘strongly agree’).

### Perceptions of risk

Participants indicated their levels of perceived risk associated with sharing health and lifestyle data with others. Participants were asked the degree to which they agreed with 12 statements about risk (rated on a five-point Likert scale from ‘strongly disagree’ to ‘strongly agree’). These statements were divided into the following categories: general risk, social risk, privacy risk, psychological risk, physical risk, and monetary risk. These categories were based on factors of a perceived risk that have been identified by previous literature as relevant to the sharing of health and lifestyle data with others.^45–47^

### Trust, Identity, Privacy and Security (TIPS) concerns

Participants were asked the extent to which particular factors relating to TIPS concerns are important when deciding whether or not to share health and lifestyle data with others. Three factors were considered for each concept of TIPS; these were selected based on previous findings from a related study conducted as part of the broader research programme^21^ and unpublished qualitative findings from our research team into the TIPS concerns of those living with LTHCs. Participants were invited to rate their importance on a five-point Likert scale ranging from ‘Not at all important’ to ‘Extremely important’). For example, about trust, we asked participants how important it is to be familiar with the recipient in order to share personal information. Questions on trust also investigated how the relevance of requested information influences trust in the recipient, as well as asking if mutual disclosure of information is important to those with LTHCs. Statements about identity concerns addressed the use of pseudonyms and investigated the perceived importance of having the option to manage digital identity and control one's online presence. Questions addressing the importance of privacy asked about the need for anonymity when sharing and the ability to select and control how personal data is shared. Finally, security questions were asked about the perceived importance of dependable data storage, the ability to manage access to personal data and the need for digital and physical safeguards to protect health and lifestyle data.

### Attitudes towards sharing – rating activities

Participants completed rating activities to indicate their willingness to share different information types with different recipient groups. These rating activities were divided into seven tasks by recipient group (HCPs, Public Health and Research, Other People with the Condition, Family, Friends, Work and Social Media). For each recipient group, participants were asked to rate their willingness to share 12 information types (contact information, a photo of themselves, demographic information, medical information, consequences of illness, mental health information, sexual health information, other health information, substance use, sleep, diet and nutrition, and exercise). The rating was conducted on sliding scales from zero (completely unwilling to share) to 100 (completely willing to share). This method was developed from previous research that used a visual analogue scale to provide a normalised measure of 0–100 to rate how comfortable a participant would feel sharing particular identity attributes in different sharing contexts.^21^

### Stigma

Participants were asked if they felt they had experienced stigma as a result of their health condition(s). If participants had previously stated that they have multiple health conditions, they were asked to indicate which of their selected conditions were relevant to their experiences of stigma. Participants’ perceived level of stigma associated with their LTHC(s) was measured using the Stigma Scale for Chronic Illness (SSCI-8).^48^ This scale rates stigma across eight items on a five-point Likert scale. Total scores range from 8 to 40, with higher scores indicating higher levels of perceived stigma.

### Information sensitivity scale

Perceived sensitivity of health and lifestyle data was measured using the Workplace Information Sensitivity Appraisal (WISA).^49^ This 17-item scale consists of five subscales pertaining to perceived information sensitivity: Privacy, Worth, Consequences, Low proximity interest by others and High proximity interest by others. This scale has been found to have strong psychometric properties and has been used to measure the perceived sensitivity of health information.^49^

### Technology preferences

Finally, the survey asked about preferences for the technological sharing of self-generated health and lifestyle data via a digital platform. This addresses part of the broader goals of the INTUIT research programme to design digital tools that remove the barriers to collecting and sharing self-generated health and lifestyle data in order to improve the health and well-being of stigmatised populations. Participants were asked their perceived importance of 11 technological features of digital platforms (usability, appearance, connectability to other devices, connectability to other applications, storage, pattern recognition, social interaction, security, access, anonymity and trustworthiness) when considering whether or not to share health and lifestyle data with others via a digital platform. Participants indicated their degree of perceived importance for each factor on a five-point Likert scale, ranging from ‘not at all important’ to ‘extremely important’.

Full details for our questionnaire are available as part of our pre-registration on the Open Science Framework (osf.io/h3mjv/).

### Analysis

All statistical analyses were performed using SPSS software version 26 with the exception of factor analysis and modelling being conducted using AMOS version 26. Analysis across measures that collected data via five-point Likert scales used Spearman's rho tests for correlational analysis. Collated scores for overall TIPS concerns, overall willingness to share and overall perceived risk were treated as continuous variables. Therefore, independent *t*-tests were used to examine differences in these variables between those who reported experiencing stigma associated with their condition and those who did not (*N*_stigma_ = 145, *N*_no-stigma_ = 105).

Factor analysis was conducted for our measures of TIPS, Perceived Risk, Perceived Stigma (SSCI-8 scale), and Perceived Sensitivity of Information (WISA scale) to determine which measures should be treated as unidimensional, and which measures reflect multiple factors. Firstly, the 12 item TIPS measure was shown to have high reliability (Cronbach's α = .84). The initial factorability of the 12 TIPS items was then examined using several criteria. All 12 items correlated at least three with at least one other item, suggesting acceptable factorability (see Supplemental Table S5). Secondly, the Kaiser-Meyer-Olkin (KMO) measure of sampling adequacy was .82 (above the recommended value of .6) and Bartlett's test of sphericity was significant (*X*^2^(66) = 1166.58, *p* < .001) suggesting the items are structurally related. Finally, the communalities were all above .49 confirming that each item shares common variance with other items. Principal components analysis was used to identify if TIPS concerns should be analysed by individual factors. Initial eigenvalues indicated that three factors explained 30%, 13% and 9% of the data variance. A single factor solution was preferred because of the ‘levelling off’ of eigenvalues after the first factor, as well as the fact that factors did not load in accordance with the theoretical categories of individual TIPS concerns. Furthermore, interpreting TIPS concerns as a single summed score follows recommendations that sum scores are most acceptable when using exploratory scales and can allow the analysis to preserve the variation of the original data.^50^

A similar approach was taken for the 12 items of perceived risk associated with sharing self-generated health and lifestyle data with others, which indicated high internal consistency (Cronbach's α = .90). All items correlated well with others (see Supplemental Table S6, KMO score = .86, Bartlett's test was significant (*X*^2^(66) = 1670.38, *p* < .001), and communalities were all above .52. A single factor solution that explained 48.76% of the variance in the data was preferred. Therefore, subsequent treatment of perceptions of risk used total summed scores of perceived risk, averaged to fit the existing scale parameters.

The SSCI-8 scale of perceived stigma was also shown to have high internal consistency (Cronbach's α = .89). All items were well correlated with each other (see Supplemental Table S7), KMO score = .88, Bartlett's test was significant (*X*^2^(28) = 1138.45, *p* < .001) and communalities were at .5 (except for 1 item). Principal component analysis extracted a single component that corresponds with previous validation of the scale as a unidimensional measure.^48,51^

Finally, the 17 items of the WISA scale were examined using confirmatory factor analysis to determine the model fit for the five previously identified factors (Privacy, Worth, Consequences, Low Proximity Interest, and High Proximity Interest; see Supplemental Figure S1 and Table S8) in accordance with the original scale construction and validation.^49^ The scale indicated acceptable internal consistency (Cronbach's α = .69). Goodness of fit for the model was determined using (1) the *X*^2^ goodness of fit statistic, (2) the Comparative Fit Index (CFI), and (3) Root Mean Square Error of Approximation (RMSEA). The hypothesised model fit produced a significant *X*^2^ statistic, *X*^2^(109) = 207.16, *p* < .001, indicating poor model fit. However, this test is often criticised for being too sensitive for sample sizes over 200.^52^ The two remaining goodness of fit statistics produced results within accepted thresholds (CFI = .92, RMSEA = .06) indicating that the five original factors should be considered a good fit to the data, in agreement with the original scale construction and validation.^49^ Therefore subsequent correlational analysis across measures treated TIPS, Perceived Risk and SSCI-8 as single dimension measures, whereas perceived sensitivity of health and lifestyle information considered a treatment of five separate factors.

## Results

### Descriptive statistics

Table 1 presents the descriptive statistics for our sample, whose ages ranged from 18–77 years (*M* = 39.20, SD = 14.78).

Demographic variables were collected to present the extent of diversity of the recruited sample. Gender showed no effect on the frequency of data recording or sharing, overall willingness to share, overall perceived risk from sharing, overall TIPS concerns, levels of perceived stigma or overall WISA scores (see Supplemental Tables S12 to 14). There was no effect of age bracket on these variables with the exception of overall perceived risk from sharing and overall WISA scores. However, post hoc analysis showed no general trend with respect to the age bracket (see Supplemental Tables S12 to 14). Therefore, the reporting of subsequent analysis and results will not discuss demographic variables.

Table 2 presents the frequencies for self-reported LTHCs reported by our sample, along with their reported primary LTHCs. The most frequently reported LTHCs were depression (*n* = 88) and anxiety (*n* = 87). All participants reported between one and nine LTHCs in total, (*M* = 2.69, SD = 1.71) and over 60% of our sample reported having lived with their LTHC(s) for more than 10 years. The most commonly reported primary LTHC was ‘Multiple LTHCs’ (*n* = 47), followed by depression (*n* = 21; see Table 2).

### Recording and sharing self-generated health and lifestyle data

Across all presented information types, the mean participant response was that they record their health and lifestyle data either ‘when the need arises’ or ‘less than once a month’. However, 75% of participants reported recording at least one information type on a daily basis. The most common daily recorded information type was ‘use of medication’ (35% of sample), followed by ‘mood’ (30%) and ‘sleep’ (28%; see Supplemental Table S1). The most commonly reported method for recording self-generated health and lifestyle data was via mobile phone (50% of sample) followed by a written diary (42%) and smartwatch tracker (18%; see Supplemental Table S2).

**Figure 1.**
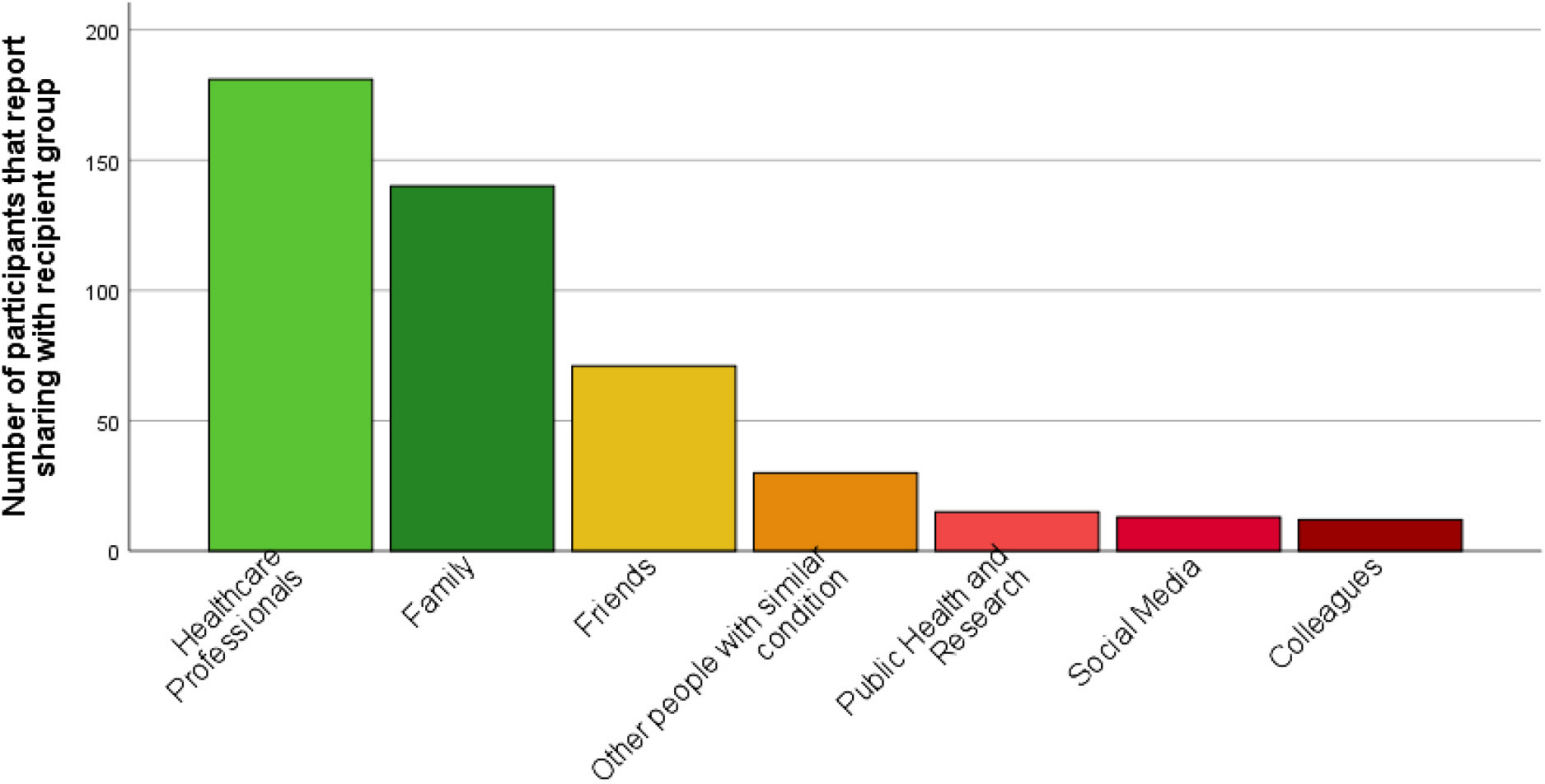
Bar chart showing the sharing of self-generated health and lifestyle data by recipient groups for participants who reported sharing with others (*n* = 202).

Of our sample, 48% reported rarely sharing their health and lifestyle data with others, 19% reported never sharing this data with others, 24% reported sometimes sharing, whereas few participants reported often or always sharing their data with others (5% and 3%, respectively). Of those who reported sharing their self-generated health and lifestyle data with others (*n* = 202), 74% reported sharing with HCPs, 60% share with family and 34% with friends (see Figure 1).

When asked about their overall perceptions and experiences of sharing self-generated health and lifestyle data with others, 42% of participants reported that they felt it was positive overall, only 8% felt it was mostly negative, whereas approximately 50% felt it was neither positive nor negative overall. Similarly, 48% of all participants felt that sharing their health and lifestyle data with others would be beneficial to them, 9% felt that it would be detrimental whereas 43% felt it would be neither beneficial nor detrimental (see Supplemental Table S3). When asked what motivates participants to share their data with others, 73% of those who reported sharing self-generated health and lifestyle data with others (*n* = 202) agreed that they do so in order to better manage their own condition and to improve their own health. Whereas, 55% of those that share agreed that they do so in order to improve the health of others. Similarly, 57% reported sharing in order to receive emotional support, whereas 49% reported doing so to provide emotional support for others. Finally, 76% of those participants who reported sharing self-generated health and lifestyle data with others agreed that they are motivated to do so in order to receive practical support from others to help manage their condition (see Supplemental Table S4). These findings indicate that not only do the majority of participants in this sample see personal data sharing as beneficial for improving their health, but also a large percentage perceived sharing with others as being beneficial for improving the health of others.

### Perceptions of risk

**Figure 2.**
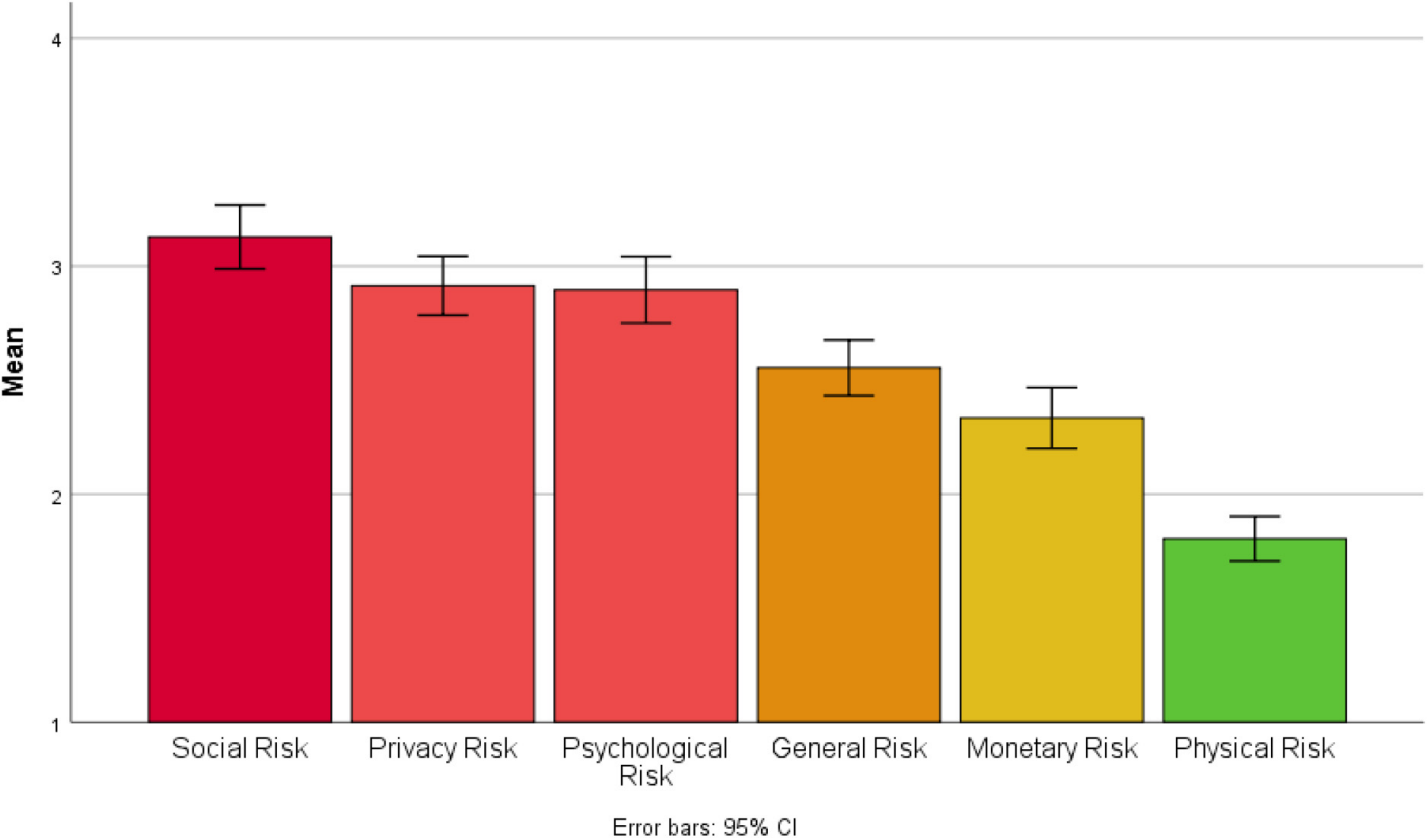
Bar chart showing mean scores for perceived risk by category.

Approximately two-thirds of our sample agreed that the benefits of sharing self-generated health and lifestyle data with others outweigh the risks. Across all categories of risk, the average participant response (mean and median) was that they ‘neither agreed nor disagreed’ that sharing self-generated health and lifestyle data posed a risk. However, sharing self-generated health and lifestyle data with others was considered to carry greater social risk and less physical risk than other categories of risk (see Figure 2). For example, 54% of participants agreed that sharing health and lifestyle data would cause others to act differently towards them.

Overall perceived risk of sharing self-generated health and lifestyle data with others was negatively correlated with both self-reported frequency of sharing with others (*r* = −.18, *p* < .01) and overall willingness to share information with others (*r* = −.24, *p* < .001). Whilst participants reported perceived benefits of sharing self-generated data for improving the health of themselves and others, they also considered doing so to be risky and potentially harmful, with significant social implications.

### TIPS concerns

**Figure 3.**
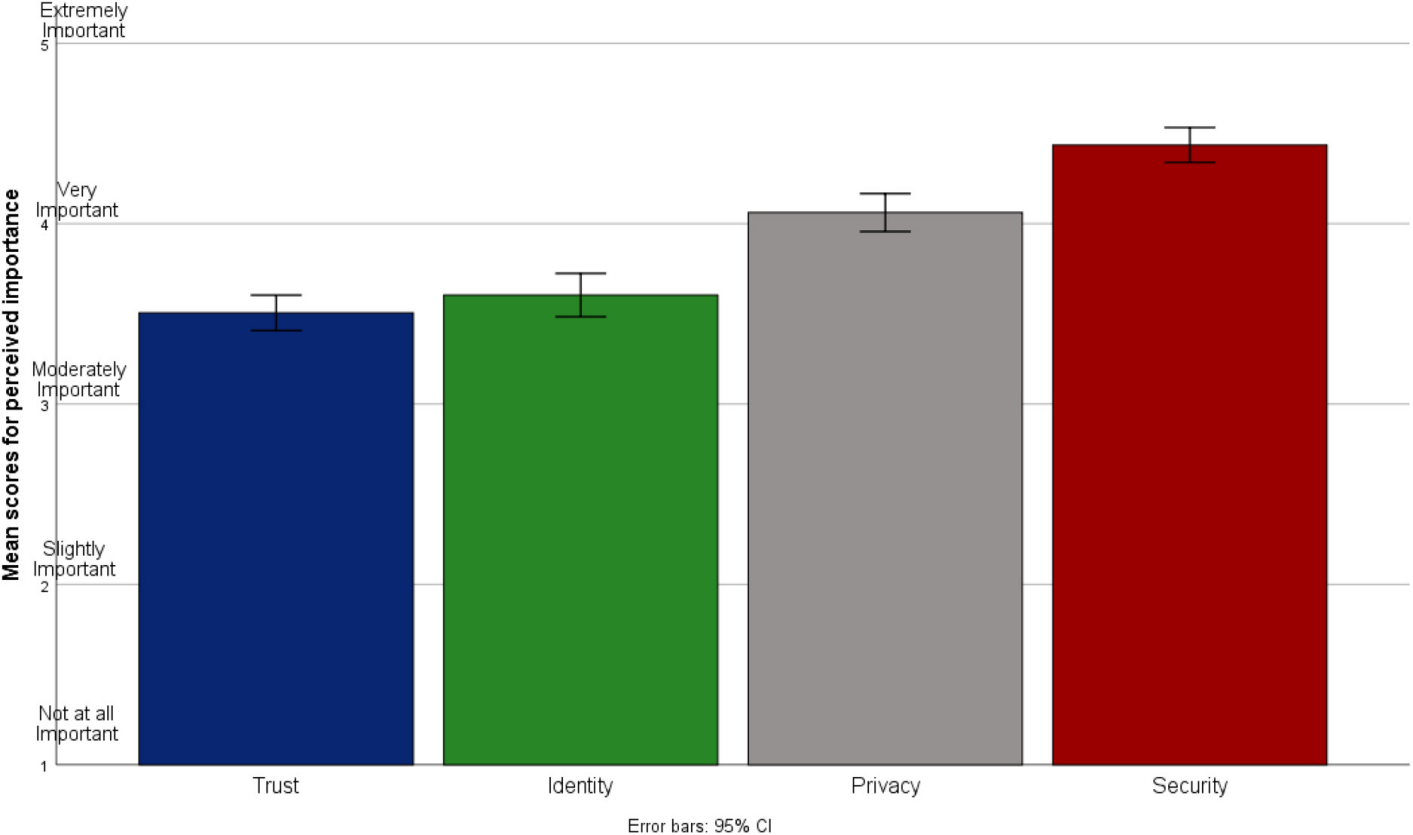
Bar chart showing mean scores for Trust, Identity, Privacy and Security (TIPS) concerns by category (*n* = 250).

Participants on average (mean and median) considered statements concerning TIPS to be ‘very important’ when deciding whether or not to share self-generated health and lifestyle data with others. Statements pertaining to the security of health and lifestyle data were considered to be of the greatest importance compared to other TIPS concerns (see Figure 3).

Overall participant TIPS scores were negatively correlated with the self-reported frequency of sharing self-generated health and lifestyle data with others (*r* = −.19, *p* < .01), as well as with overall willingness to share data with others (*r* = −.16, *p* = .01).

### Attitudes towards sharing self-generated health and lifestyle data with others

**Figure 4.**
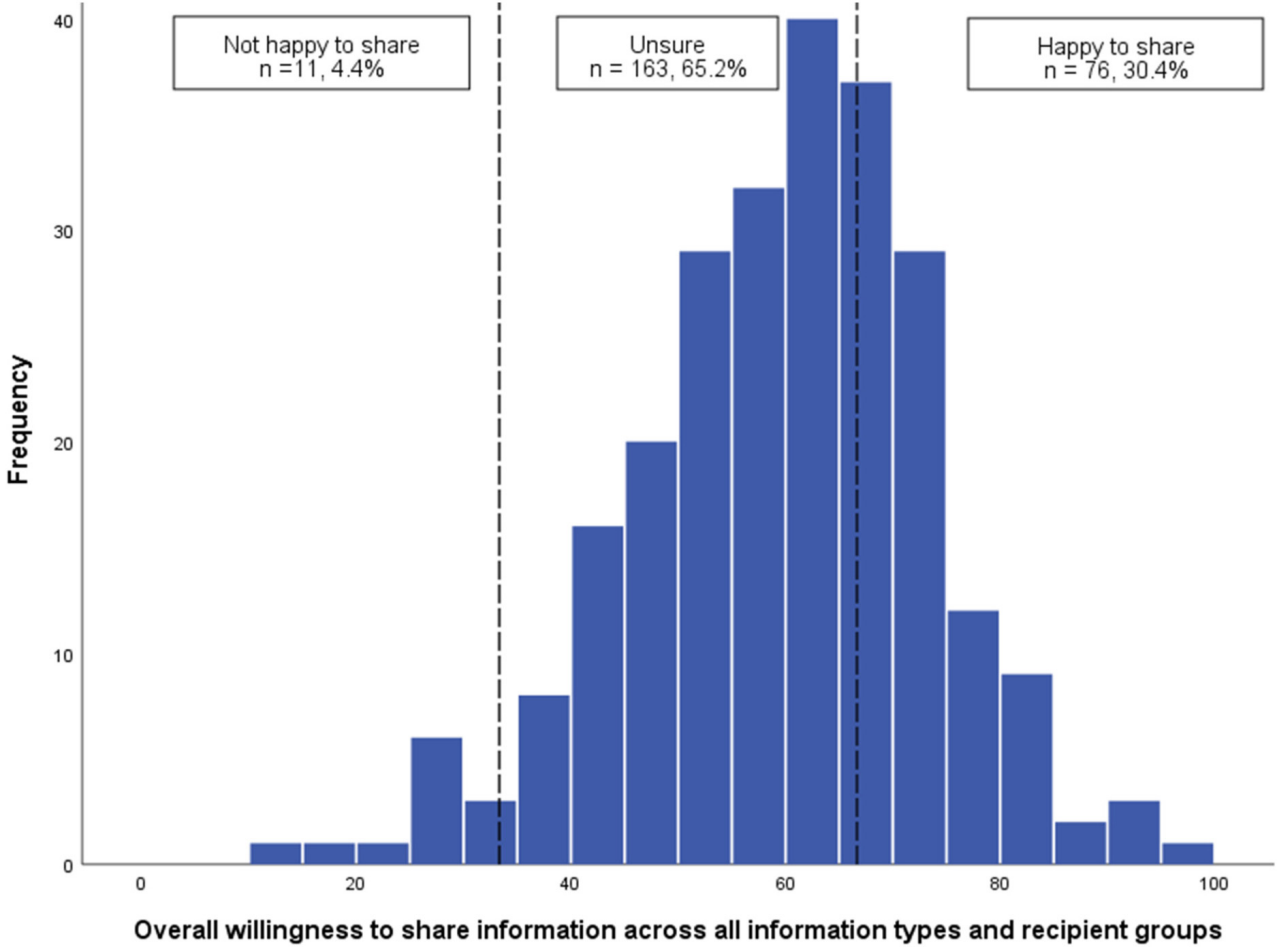
Histogram showing the distribution of overall willingness to share information across all information types and recipient groups (*n* = 250).

**Figure 5.**
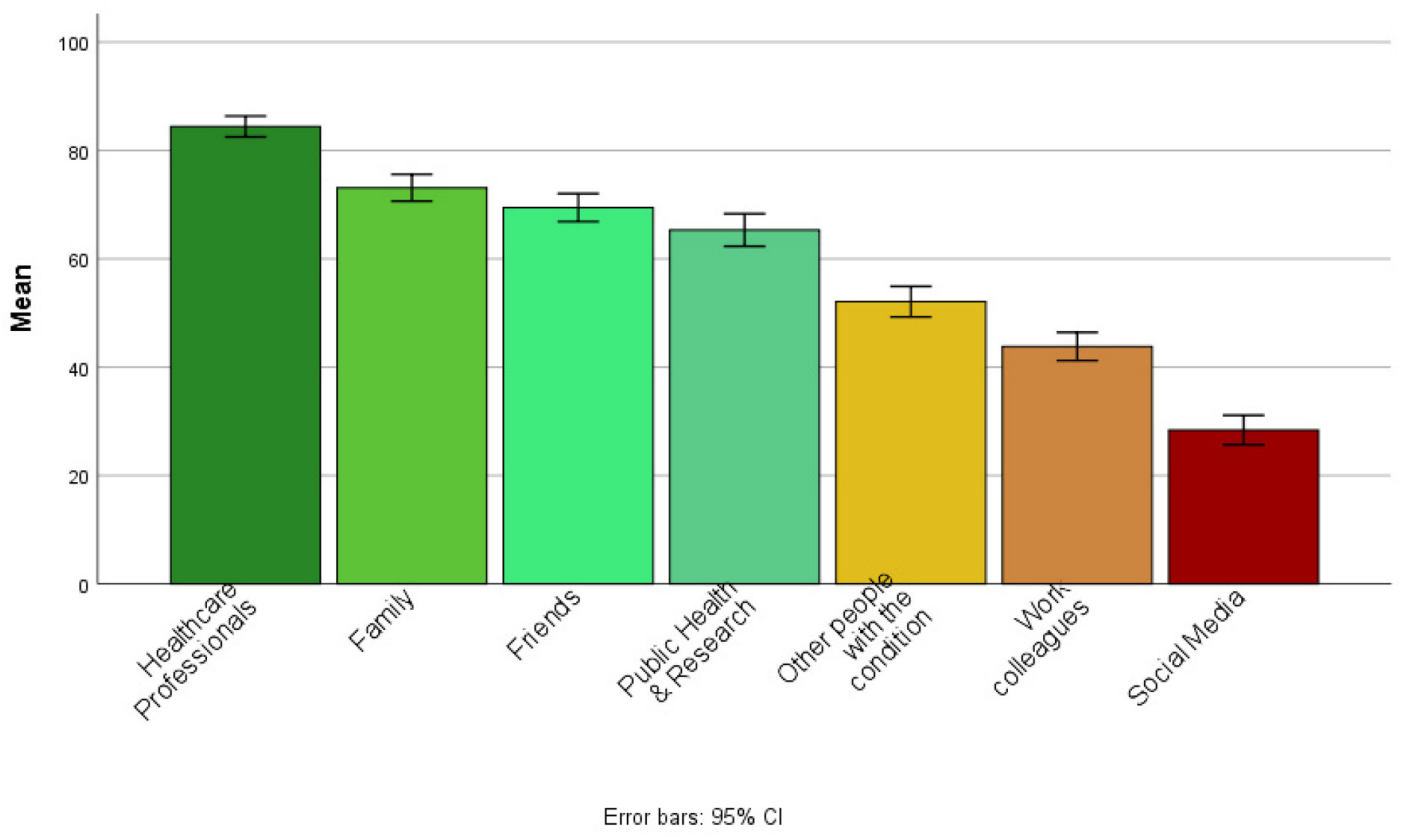
Bar chart showing mean willingness to share self-generated health and lifestyle information with others, by recipient group.

**Figure 6.**
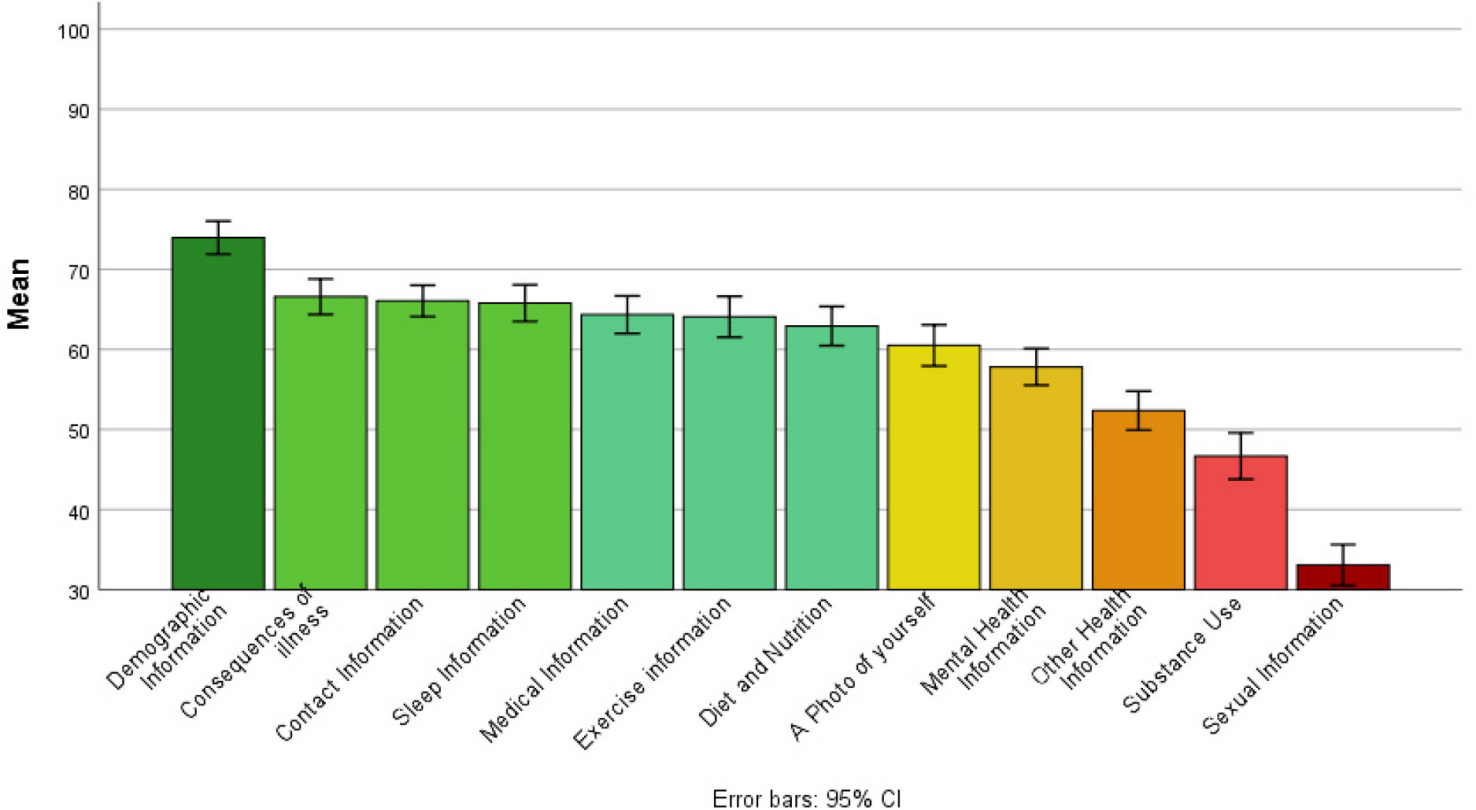
Bar chart showing mean willingness to share self-generated health and lifestyle information with others, by information type.

The mean score for overall willingness to share across all information types and recipient groups was 59.51, SD = 14.12 (0 = not willing to share, 50 = unsure, and 100 = yes, willing to share) suggesting that participants were generally unsure about sharing their self-generated health and lifestyle data with others (see Figure 4). For the recipient group, the greatest willingness to share was reported for sharing with HCPs (*M* = 84.42, SD = 15.49) and the lowest for sharing via Social Media platforms (*M* = 28.40, SD = 21.73; see Figure 5). For information type, the greatest willingness to share with others was reported for sharing demographic information (*M* = 72.95, SD = 16.57) and lowest for information of a sexual nature (*M* = 33.07, SD thinsp;= 20.55; see Figure 6).

### Stigma

Of our sample, 58% reported feeling that they had experienced stigma as a result of their LTHC(s). Most notably, 51% of those who reported suffering from anxiety (44 out of 87), 63% of those with depression (55 out of 88), and 66% with a mental health condition (23 out of 35) felt they had experienced stigma because of having their condition (see Supplemental Table S9). There was no difference between those who reported experiencing stigma as a result of their LTHC(s) and those who did not with respect to the frequency of data sharing (*t*(248) = .21, *p* = .83), overall willingness to share with others (*t*(248) = .23, *p* = .77) or overall TIPS concerns (*t*(248) = 1.32, *p* = .19). However, those who reported experiencing stigma associated with their condition did report higher levels of overall perceived risk from sharing self-generated health and lifestyle data with others (*t*(248) = 4.91, *p* < .001) and higher overall WISA scores for perceived information sensitivity (*t*(248) = 3.47, *p* < .001). Similarly, there was a strong positive correlation between levels of perceived stigma, measured by the SSCI-8, and perceptions of risk associated with sharing self-generated health and lifestyle data with others (*r* = .45, *p* < .001).

### Perceived sensitivity of health and lifestyle data

Total WISA Scale scores were positively correlated with greater total TIPS concerns (*r* = .29, *p* < .001) as well as with overall perceived risk from sharing self-generated health and lifestyle data with others (*r* = .34, *p* < .001), indicating that greater perceived sensitivity of health and lifestyle data is associated with higher perceived risk and concerns about TIPS. From the individual WISA factors of perceived sensitivity of health and lifestyle data, scores for perceived privacy of data were negatively associated with both self-reported frequency of sharing with others (*r* = −.19, *p* < .01) and overall willingness to share data with others (*r* = −.17, *p* < .01). Scores for perceiving health and lifestyle data as humiliating, embarrassing, discreditable or compromising (the ‘consequences’ factor from the WISA scale) were strongly associated with overall perceived risk from sharing health and lifestyle data with others (*r* = .52, *p* < .001). Furthermore, ‘consequences’ was the only factor from the WISA scale to be significantly higher in those participants who reported experiencing stigma associated with their condition (*M*_stigma_ = 2.77) compared to those who did not (*M*_no-stigma_ = 2.10; *t*(248) = 6.26, *p* < .001). Finally, those who perceived their health and lifestyle information as being of interest to their friends and family (the ‘high proximity interest’ factor from the WISA scale) reported more frequent sharing with others (*r* = .21, *p* < .01) and greater overall willingness to share with others (*r* = .38, *p* < .001). For full correlational results for the WISA scale, see Supplemental Table S10).

### Technology preferences

When asked about the importance of proposed features for sharing health and lifestyle data via a digital platform, the highest mean scores of importance showed a preference for platforms that are trustworthy (*M* = 4.79, SD = .50; 0 and 5 representing ‘not at all important’ and ‘extremely important’, respectively) and platforms that store data securely (*M* = 4.78, SD = .54). Whereas, the features perceived to be of least importance were allowing users to store handwritten data (*M* = 2.18, SD = 1.26) and allowing users to interact socially with others via the platform (*M* = 2.44, SD = 1.22; see Supplemental Table S11).

## Discussion

Our findings suggest that a number of factors influence both the frequency of sharing and overall willingness to share self-generated health and lifestyle data with others by people living with LTHCs. The degree to which issues concerning TIPS are considered to be important was negatively associated with frequency of sharing and overall willingness to share. Secure storage, access and the presence of safeguards to protect health and lifestyle data were reported to be the most important of all TIPS issues. Furthermore, the greater perceived risk associated with sharing self-generated health and lifestyle data with others predicted a lower frequency of sharing and overall willingness to share. The potential for harm to one's social relationships was considered the most pressing risk associated with sharing self-generated health and lifestyle data with others. The proposed recipient and information type were also found to influence willingness to share. Participants were most willing to share with HCPs and least willing to share via Social Media. For information type, participants reported being most willing to share demographic information and least willing to share any information of a sexual nature. Finally, with respect to the perceived value and sensitivity of information, the extent to which health and lifestyle data was believed to be of value to close friends and family was positively associated with increased sharing frequency and willingness to share. We discuss the implications of these key findings and make suggestions for the future design of digital platforms that look to facilitate the sharing of self-generated health and lifestyle data.

### Those with LTHC(s) report high levels of data recording but low levels of sharing with others

Three-quarters of our sample reported recording information about their health and lifestyle on a daily basis, with the most common method of data collection being via mobile phone. This is unsurprising given the recent proliferation of mobile health apps online with more than 250,000 available for download on smart devices.^53^ However, despite high levels of self-recording of health and lifestyle data, two-thirds of our sample reported never or rarely sharing this information with others. Although participants were broadly willing to share health and lifestyle data with their HCPs, they were mostly unsure about whether or not to share for public health surveillance and research. Integrating self-generated health and lifestyle data into public health work is a widespread aspiration internationally.^54^ Data from mobile devices, health trackers and handwritten journals have the potential to document longitudinal health information not ordinarily captured by routine health consultations, and identify causal pathways in health not yet considered.^55,56^ These new data have significant potential for bridging the gap between a patient's life in and outside of a doctor's consultation room, as well as to empower patients to better manage their health.^57^ Participants who reported sharing self-generated health and lifestyle data with others were most motivated to share health and lifestyle data by the potential to receive practical support from others to help manage their condition. This may include receiving assistance to complete daily activities or extra support in fulfilling work and caring responsibilities when an individual's symptoms make these difficult to manage. Given the range of potential benefits for both individual patients and public health, as well as the reported motivations for sharing data with others, it is critical that we more fully understand the barriers to effective sharing, particularly with HCPs.

### TIPS concerns are very important when deciding whether to share with others, with security being the most important

Overall, participants considered TIPS concerns as being ‘very important’ when deciding whether or not to share self-generated health and lifestyle data with others. This supports previous research that found concerns relating to issues of TIPS to strongly influence the sharing of health data via Internet-enabled technology.^21–24^ Our investigation into the perceptions of those living with a broad range of LTHCs supports the findings of previous research from our broader research programme into TIPS considerations that people living with HIV make when sharing data with each other.^21^ Specifically, TIPS considerations are very important to both those with HIV and those living with a range of LTHCs when deciding whether or not to share health and lifestyle data with others. Furthermore, deciding to share is often dependent on the context of the sharing, the type of data being shared, and the proposed recipient.

Positive associations were found between the increased perceived importance of TIPS concerns when sharing data with others, and lower frequency of sharing and lower overall willingness to share. This suggests that those with heightened TIPS concerns may be less willing to share self-generated health and lifestyle data with others. Out of the four separate components of TIPS, security concerns were considered most important. Previous findings have suggested that patients in the UK are often worried about the ability of the NHS and public health to guarantee the security of personal health data.^58^ Underlying concerns for the security of personal information have been specifically reported by those living with stigmatised LTHCs.^59^ Security was also reported as a priority when our sample was asked about which features of a digital platform (such as a mobile app) they thought would be most important for encouraging them to share self-generated health and lifestyle data with others. Again, this supports the findings of research into TIPS concerns of those living with HIV when sharing data with each other. Previous research found that participants wanted tight security measures ‘akin to banking apps’ and strict identity verification in order to facilitate the sharing of health and lifestyle data.^21^

### Heightened perceptions of risk reduce willingness to share

Those who perceived greater risk associated with sharing their health and lifestyle data with others reported a lower frequency of sharing and were generally less willing to share health and lifestyle data with others. Of the presented categories of risk (general risk, social risk, privacy risk, psychological risk, physical risk and monetary risk) social risk was considered to carry the most weight with over half of participants agreeing that sharing health and lifestyle data would likely cause others to act differently towards them. ‘Social risk’ refers to the potential to lose one's standing in a societal group.^45^ Our results suggest that many of those living with LTHCs believe that sharing certain aspects of their health and lifestyle data would alter the dynamics of their relationships with others. This may help to explain our finding that having the ability to socialise via a digital health data sharing app was described by our sample as one of the least preferred features. Given that the greatest degree of reported concern was for social risks, future studies may look to investigate specific social fears and to explore ways of mitigating the perceived risks associated with potential damage to social relationships. Furthermore, research may look to investigate concerns about social risks in the context of sharing between patients and HCPs; a context where sharing may be considered to pose less of a threat to one's social relationships than sharing with family, friends, colleagues and those living with a similar LTHC.

### Perceived sensitivity of health and lifestyle data

Perceived sensitivity of health and lifestyle data overall was positively associated with TIPS concerns, suggesting that the more sensitive those living with LTHCs believe their health information to be, the more concerned they are about TIPS when considering whether or not to share their data with others. From the specific factors of what participants believe makes their data more sensitive, those who perceived their health and lifestyle information as being of interest to their friends and family reported more frequent sharing with others and greater overall willingness to share their data. Family and broader social support have been highlighted as playing a key role in managing LTHCs, suggesting a positive relationship between social support and chronic illness self-management.^60,61^ Consolidating our results, we suggest that understanding your health information to be of value and interest to those around you makes you more likely to share self-generated health and lifestyle data with others. Given the discussed potential benefits for health and care management, this key finding highlights the important role that family and close social networks can play in promoting the effective sharing of data and helping to manage LTHCs.

### Those experiencing LTHC-related stigma reported higher levels of perceived risk associated with sharing

Of our sample, 58% reported experiencing stigma as a result of their LTHC(s). Most notably, more than half of participants with anxiety, and roughly two-thirds of those suffering from depression or other mental health conditions reported having experienced stigma in relation to their LTHC. This supports an established body of literature suggesting that despite improvements to mental health awareness in recent years, experiences of stigma continue to be reported by those who manage mental health conditions.^33,62,63^ Reports of experienced stigma among those living with LTHCs are concerning given that such experiences of stigma may have a detrimental impact on health and lead to delays in seeking diagnosis and treatment.^32,64,65^ Contrary to our registered predictions, those who reported experiencing stigma associated with their condition did not report a lower frequency of sharing compared to those without experiences of stigma. That said, it is possible that this may be explained by the overall low levels of frequency of sharing health and lifestyle data with others reported by our sample. It should also be noted that our sample did not include anyone living with HIV. HIV is typically associated with experiences of stigma,^21,37,66^ therefore further research may look to directly compare the experiences and perceptions of stigma reported by those living with HIV, with those living with different LTHCs. However, from our sample, those with experiences of LTHC-related stigma were more sensitive to the potential for negative consequences as a result of sharing health and lifestyle data with others, and reported higher levels of perceived risk. These negative consequences are related to the potential for humiliation and social embarrassment which suggests that experiencing stigma associated with your LTHC may make you more fearful of the potentially harmful social consequences from sharing your health and lifestyle data with others. There was also a strong correlation between perceptions of risk associated with sharing and perceptions of condition-related stigma, suggesting that beliefs around stigma are closely related to perceptions of risk.

### Designing digital platforms for sharing self-generated health and lifestyle data with others

Our study delivers a number of key findings that may inform the design of digital platforms for sharing self-generated health and lifestyle data with others by those living with LTHCs. Firstly, the high degree of self-recording of health and lifestyle data via digital devices, combined with the generally reported belief that sharing this data with others can be beneficial, suggests that there is potential for widespread sharing via digital platforms, provided that key barriers to sharing can be overcome.

Our findings suggest that digital platforms that highlight the secure storage, access and presence of digital safeguards to protect self-generated health and lifestyle data may enhance trusted sharing. This was further emphasised by issues concerning security being considered the most important individual TIPS area by people living with LTHCs. Additionally, overall willingness to share via digital platforms may be affected by the categories of information that are requested. People with LTHCs reported a general willingness to share demographic data, but were least willing to share information of a sexual nature. Therefore, digital platforms that provide individuals with control over which categories of information are both recorded, requested and shared may help to enable the trusted sharing of self-generated health and lifestyle data.

Participants reported the greatest willingness to share with HCPs and were most motivated to share by the potential to improve their health and receive practical support to better manage their condition(s). Digital platforms that emphasise the practical benefits of sharing self-generated health and lifestyle data may encourage increased sharing. This may be achieved by digital platforms presenting users with practical examples of how self-generated health and lifestyle data is used to facilitate improved diagnosis, treatment and delivery of care. Our findings also suggest that demonstrating to the users of such digital platforms how this data could be used to improve the health of others may also enhance trusted sharing. Participants were least willing to share self-generated health and lifestyle data via social media. This suggests that digital platforms designed for the sharing of health and lifestyle data that also look to facilitate broader connections via social media may be ineffective in encouraging sharing. People may want platforms for sharing their *data* that remain separate from those that support more social interactions. Indeed, the reported technological preferences of our sample indicate that the ability to interact socially via a digital platform for sharing health and lifestyle data is considered to be of little value. This may be due to a general distrust in social media and speaks to the previously discussed concerns about the potential for social harm as a result of sharing self-generated health and lifestyle data with others.

### Limitations and future work

Our sample reported living with LTHCs that were widely distributed across more than 50 different categories of health conditions. Despite adding to the richness and diversity of our sample, due to the small number of participants for each health condition, we were unable to draw meaningful comparisons across different LTHCs. Further research may look to target specific LTHCs of interest to investigate differences between conditions in attitudes towards sharing self-generated health and lifestyle data with others. This will help to determine the extent to which the perceptions and experiences of specific groups differ from the broader category of those living with LTHCs with respect to the sharing of self-generated health and lifestyle data with others. An additional limitation concerning our sample relates to our use of an online recruitment platform, through which participants had already elected to share personal information such as their age, gender, ethnicity and health status. It is possible that participants recruited via this platform may be more willing than the broader UK population to share self-generated health and lifestyle data with others, introducing a potential bias.

In addition, the most commonly reported primary care need of our respondents was living with multiple LTHCs. Previous research has suggested that living with multiple LTHCs can threaten one's self-image and identity, lead to experiences of stigma and impaired quality of life.^67,68^ A recent review examining digital interventions for people living with multiple LTHCs highlighted that there is still little evidence for successful health information technology solutions that improve care for those living with multiple conditions.^69^ Given the increasing normality of living with multiple LTHCs, understanding more about the ways in which people with multiple conditions consider and manage their digital health will also impact upon the design of technological solutions to improve support overall.

Finally, future research may look to examine attitudes towards the automatic and unintentional sharing of data with the providers of digital platforms and devices. Many users have little knowledge of how their data is used and shared. A recent literature review suggested that a lack of attention has been given to understanding attitudes towards the sharing of health and lifestyle data with third parties, which suggests the need for future study.^70^

## Conclusion

Despite those living with LTHCs reporting high levels of daily recording of health and lifestyle data, these data are rarely shared with others. Those with LTHCs are most willing to share with their HCPs, but the overall low levels of sharing suggest a potential missed opportunity for public health professionals to gather valuable information that may provide key insights for improving care at a population level. Personal security concerns were found to present the greatest barrier to sharing; and security has been highlighted as a key desired feature for digital platforms that facilitate the sharing of health and lifestyle data with others. This has direct implications for the design of digital tools that look to facilitate the effective sharing of self-generated health and lifestyle data, and suggests that prioritising dependable security features is likely to encourage sharing. Experiences and perceptions of stigma as a consequence of a person's condition(s) were strongly associated with increased levels of perceived risk relevant to sharing personal health and lifestyle data with others. Participants were most concerned about the potential harm that may be caused to one's social relationships as a result of sharing health and lifestyle data with others. This has implications for the design of digital platforms aimed at facilitating the sharing of self-generated health and lifestyle data and suggests that features that look to incorporate broader sharing via social media may be ineffective in enhancing data sharing. The findings of this study offer strategic considerations for further focused digital health research to address data security concerns in the enhanced use of self-generated health and lifestyle data, and to understand the perceived risks and negative consequences associated with data sharing. Addressing these concerns will be necessary to overcome current barriers and to encourage the effective sharing of self-generated health and lifestyle data by those living with LTHCs.
